# Microbiome Yarns: bacterial predators, tissue tropism and molecular decoys

**DOI:** 10.1111/1751-7915.13706

**Published:** 2020-12-26

**Authors:** Kenneth Timmis, Franziska Jebok, Gabriella Molinari

**Affiliations:** ^1^ Institute of Microbiology Technical University Braunschweig Germany; ^2^ Uilenstede 510 Amstelveen The Netherlands; ^3^ Central Facility for Microscopy Helmholtz Centre for Infection Research Braunschweig Germany

## Abstract

This *Crystal Ball* speculates on the potential of molecular decoys for prevention and therapy in infectious diseases. It is dedicated to the memory of Singh Chhatwal, who pioneered research on disguises and decoys produced by *Streptococcus*, and so much more.

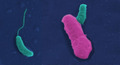

**Part 1** ^1^, ^2^, ^3^, ^4^ ***: Southend Greyhound Stadium, Essex, September 6, start of the 9th race of the evening.***

***Commentator:*** Well ladies and gentlemen, all the dogs are in their boxes, so we’ll be starting the 9.20 race in a few moments……………. *And they’re off!* Oh, what is that? *Del’s Dasher*, the 5‐2 odds‐on favourite, and *Sid’s Streaker*, the 2‐1, have stumbled out of their boxes and are now lying on their backs wagging their tails.

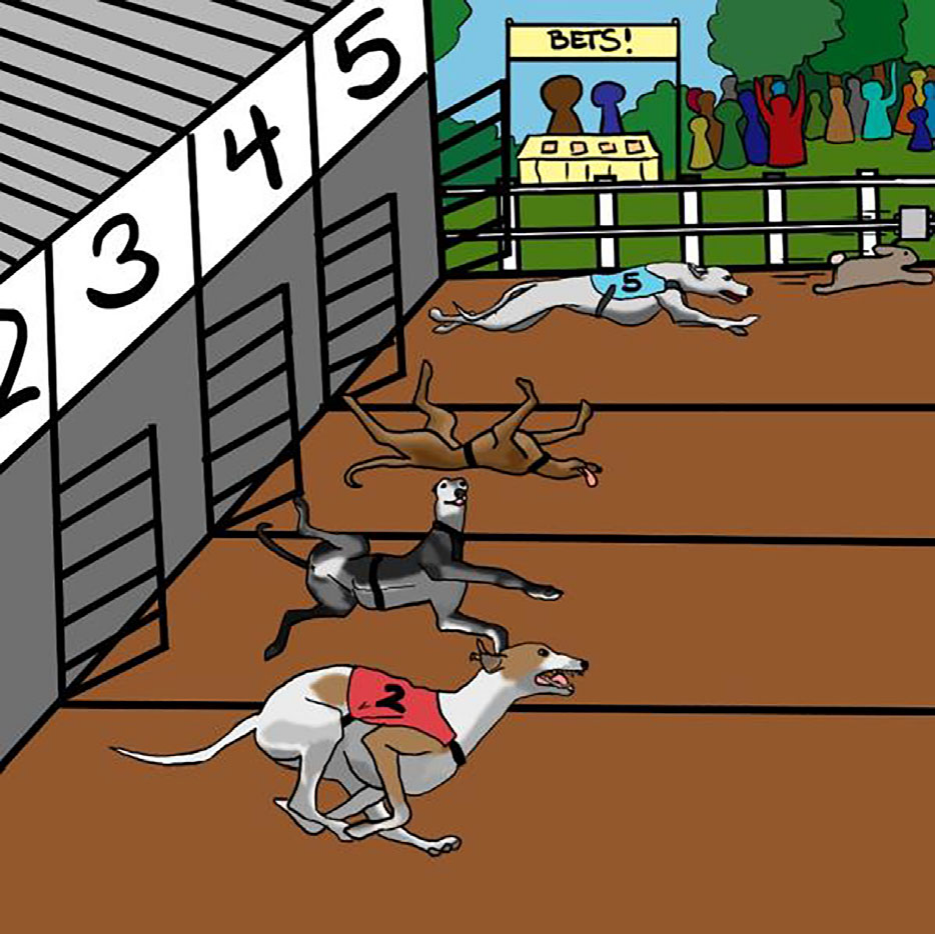

Looks as though they have caught the *silly behaviour syndrome* that affected one of the dogs last week. But look at number 1 streaking ahead………………oh well done! *Batoutahell* at 7‐1 won the race well ahead of the pack! Well, this was an exciting race but would definitely have been even more so, if the favourite had been able to compete. *SBS* is turning out to be a real issue for our sport.

*15 min later: Derek (Del) Whippet and Sidney Pointer, the owners of the dogs affected with SBS, are distractedly fondling their animals, which are responding enthusiastically, though behaving as if they were somewhat inebriated, and manically scratching their ears.*

***Del:*** Sid: this is obviously a nobble by the Moriarty Syndicate, a repeat of the favourite‐tampering we saw last week. And a complete disaster: I heard on the grapevine that *SBS* might be infectious and, if so, could affect our entire kennels!

***Sid:*** I know, Del: what can we do?

***Del*:** Apparently not much: the vet my kennel uses said that the *SBS* bug infects a part of the brain that is not accessible to drugs currently available for dogs, and anyway the bug is apparently pretty drug resistant. We’ll have to have the dogs put down and hope that the infection has not already spread to others.

***Sid:*** Hells bells! These dogs represent years of breeding and training, and investment of all my meagre earnings, quite apart from future prize money. And, even more importantly, they are family! I think I'll go to the pub over the road and drown my sorrows.

***Del:*** Hang on a minute: you just gave me an idea. An acquaintance of mine, Jilly Deels^5^, a canny microbiologist from Essexhoe University who knows almost everything worth knowing, lives nearby and is a regular of that pub. We can see if she is there and ask if she has any ideas.

***Part 2: The Hound and Hare, a favourite watering hole of the dogtrack community***

*20 minutes later, in front of the Hound and Hare, just before last drinks time.*

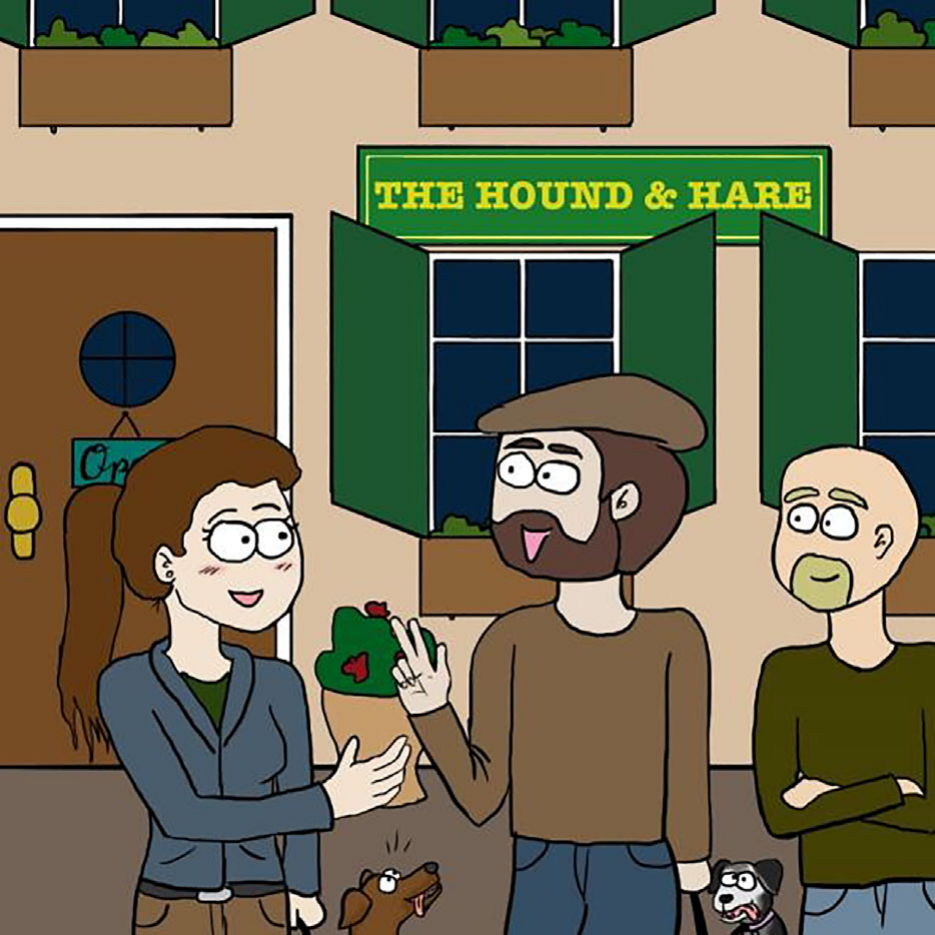

***Del:*** Hello Jill, nice to see you again! This is Sid, a fellow dog trainer.

***Jilly:*** Hello fellas – I’m going to drown my sorrows: Southend United lost against Brighton and Hove Albion: it was a really close match!

***Sid:*** Oh hell, yet more bad news: I had a tenner riding on the *Shrimpers*! Let’s go inside and I’ll get the drinks in: what’ll you have, Jill? Del: is yours the usual or something a bit stronger?

***Del:*** Well: seeing as it is close to last orders, I’ll have the usual plus a large scotch.

***Jilly:*** Thanks Sid: I’ll have a glass of bio‐Carménère

***Sid:*** Evenin Ernie: I’d like a pint of Namad's Narrowside, an IPA, a large glass of bio‐Carménère, two double scotches, and one for yourself.

*After they all take a longish, satisfying sip and, in one case, a discrete burp:*

***Del:*** Jill, we have a serious problem and wondered if you had any suggestions. These two dogs have probably got *SBS* and, apart from the misery of having to put them down, there is the risk that the infection may have spread to the others in the kennels. Is there anything we can do about it?

***Jilly****, looking very sympathetic as the dogs nuzzle her affectionately, and distracted while considering the problem:* ………………Well fellas ‐ you sometimes need a bug to fight a bug: this looks like a job for *Super Bdello*. After closing time, we’ll walk these lovelies round to my place and see what can be done.

*30 min later in Jilly’s ecohouse on the outskirts of Southend.*

***Jilly:*** Come in lads. Take a seat on the reclaimed wood benches over there. What would you like to drink? I do a mean homemade sloe gin‐elderflower cordial cocktail, or would you prefer a cup of fair‐trade tea? Both tea? Low fat milk? Non‐refined sugar? Two spoons? Ok!

Now to business: Now to business:

we obviously need to stop this bug in its tracks. Super Bdello is a bug I have designed from a bacterium called *Bdellovibrio* ^6^

that invades other bacteria and eats them from the inside^7^.

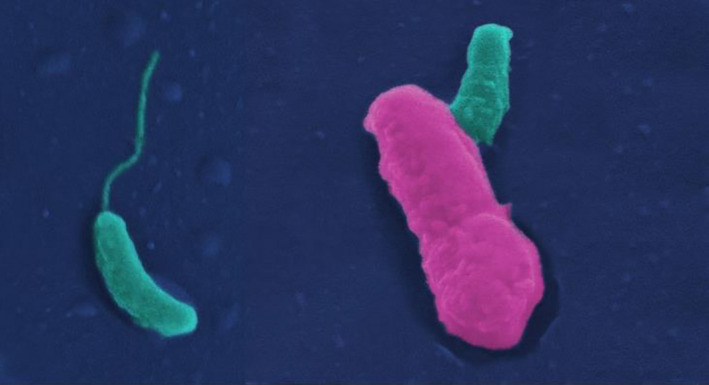

***Sid****, looking decidedly squeamish:* Oohh, sounds horrible!

***Jilly****, enjoying the attention ***:**** The trick will be to get *Super Bdello* across the blood:brain barrier to the site of the infection. To do this, I’ll equip it with the equivalent of a satellite navigation system to enable it to find its way to the target *SBS* bugs, which it’ll gobble up in next to no time. This is an agar plate of my little attack troop, feeding on *E. coli*, a gut bacterium we all have inside us. It is in perfect condition to rapidly gobble up any *SBS* bugs that it can hunt down. I call it *Super Bdello* because I have engineered into it a number of new features that make it a particularly effective anti‐bacterial agent.

***Del:*** What amazing things you microbiologists keep in your kitchens!

***Jilly:*** Right, I’m just harvesting *Super Bdello* and introducing it into the activated smart microcapsule fabrication unit, to create microcapsules containing *Super Bdello* ^7^. Now, the trick: I am looking in my spice rack of microbial reagents…..ah, here they are – freeze‐dried PilA^8^ protein and Srr glycoprotein^8^ – we’ll give a couple of generous shakes, now a bit of a swirl, and that’s it. These proteins coat the activated microcapsules and, after introduction into the blood stream, will enable microcapsule attachment to and transfer across the blood:brain barrier. In other words, when injected, the microcapsules will home in on the blood:brain barrier, cross into the brain, and release *Super Bdello*, which will then charge towards any inflamed tissue and gobble up any bacteria it finds.

Ok: now hold your beauties still and I’ll gently inject the microcapsules into their ear veins…….. Well done, my lovelies, all over! Right: to make sure that the treatment does not have any side effects, you better leave the dogs with me so that I can keep an eye on them. My guess is that the *SBS* will be gone in 8h, but the dogs better stay for 3 days, just to be sure. I will, however, have to shut them away from the predatory feral cats I have adopted, which can be quite mean to good‐natured dogs like these.

***Sid:*** Many, many thanks, Jill. If this works, you’ll have my undying gratitude. If any of the others in the kennel go down with *SBS*, can I bring them to you?

***Jilly:*** Yes of course. *Super Bdello* loves a challenge.

***Del:*** Yes, that is really a relief! But what can we do about future nobbling by the Moriarty Syndicate? Jill: do you have any ideas?

***Jilly:*** Well: that is a bit of a tall order. I think we'll need help from a second cousin of mine, James Bondage^9^ from MIH57^10^ – his job is to protect British interests and way of life from those who would sabotage either one, and he’s always eager to help folk in distress, especially if he can queer the pitch of some villains at the same time.

***Del:*** Oh, it would be terrific if Bondage could be persuaded to help us out!

***Part 3: Five months later: Friday evening,*** ***5.35pm, in the main bar of the Bulls and Bears in the City (London), a favourite watering hole of the Financial Masters of the Universe*** ^11^ ***(which, because of the obscenely high bonuses paid out to some of its customers each year, has a remarkable stock of alcoholic beverages) and would‐be FMUs. James Bondage of MIH57 and Mamba von Spectre^9^ of Menacyn^9^ face off warily:***

***von‐Spectre****, nonchalantly leaning against the bar:* Good evening James. It is wonderful to see you again. To what do I owe the pleasure of your kind invitation? Oh: but I neglect common curtesy ‐ do forgive me ‐ what shall we drink at the expense of Her Majesty’s Government?

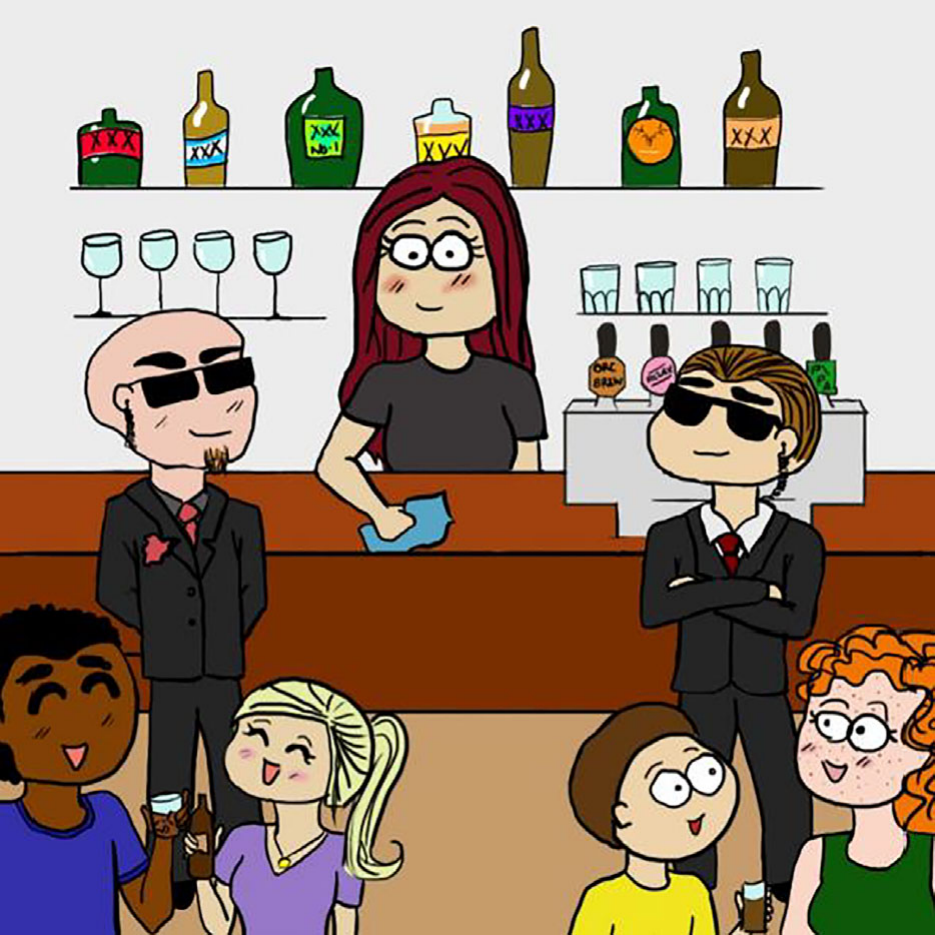

***Bondage:*** Hello Mam. I am fine, thank you. Well: seeing as Autumn is here and the nights are drawing in, I’ll have a glass of Namad's Narrowside.

***von‐Spectre:*** Miss: a pint of Narrowside for my thirsty friend here and a generous shot of that rather delectable and obscenely expensive WBV^12^ for me! Oh no: let’s have a bottle; HMGov can afford the several grand it costs: please bring the drinks over to the corner table.

***von‐Spectre****, after downing 4 fingers of WBV:* Now James: I am all ears.

***Bondage:*** *after a long, slow, satisfying draw on his glass*: Ok Mam ‐ here’s the thing. It has come to the attention of MIH57 that there is systematic sabotaging of racing greyhounds by the Moriarty Gambling Syndicate, one of the many criminal enterprises of your employer, Menacyn.

***von‐***Spectre*:***** James, I have no idea where MIH57 acquires its intelligence these days ‐ perhaps from the retired miners who frequent British dog race tracks. But, though the idea is certainly an attractive notion for, let’s say, creative enterprises, it is all new to me.

***Bondage:*** Well: of course you would say that, so let me provide a bit more background to channel us towards a meaningful dialogue. A few months ago, some dogs were deliberately infected with a pathogenic bacterium causing silly behaviour syndrome, or *SBS*, and of course could not perform well. Fortunately, they were cured by the prompt intervention of a brilliant British microbiologist and are back on form again, winning races. Unfortunately, it is rather unsatisfactory to have only a treatment/response mode course of action, since the logistics are challenging, and gambling manipulation prior to treatment cannot be prevented. So HMGov implemented a pro‐active solution. We have now spiked your bioterrorism gun and wanted to let you know, so that both HMGov and Menacyn can avoid an unnecessary wastage of more resources on this unsavoury business. You have obviously spent a goodly amount of your ill‐gotten gains on developing *SBS*, its mode of delivery, and an effective vaccine against it to protect the dogs you bet on, as have we in developing counter‐measures.

***von‐***Spectre*:***** James, this is certainly an interesting fairy tale you spin, but I repeat: Menacyn has no activities in this arena. However, since you are such a good friend, and HMGov is so courteous in picking up the tab tonight, if you provide me with a few details of the spiking, I might have a word in the ear of a colleague in my organisation. Perhaps someone else knows something and can help you direct your efforts more productively.

***Bondage:*** Right. The story goes like this. A while ago, Menacyn microbiologists learned of a new discovery, namely that a certain human gut bacterium, called *Casanovia resplendenti*, or CNR for short, produces a testosterone‐like compound, or TLC.^13^ Providing CNR in a probiotic preparation increases the amount of TLC produced in and absorbed by the gut, with the result that muscle mass and athletic performance increases. So, what your unethical scientists did was to engineer the TLC production genes into a greyhound gut‐colonising bacterium, and feed it as a probiotic to would‐be champion greyhounds. The, for Menacyn, good news is that the probiotic‐fed hounds were faster on the track. The, for Menacyn, bad news was that, dogs being dogs, and relishing the sausages left by others during morning walkies, the nice engineered bacteria rapidly spread to other dogs which then became as fast as those initially treated, so all benefit evaporated.

***von‐Spectre****, downing another 4 fingers:* James: this is all very fascinating but taking rather a long time. Could you please get to the spiking point. *MISS: another bottle!*

***Bondage****:* Hhmm: doesn’t Menacyn pay you enough, or is it just that you must take every opportunity to get one over on MIH57 in retaliation for all your devious enterprises we headed off at the gulch? *MISS: at this point, I’d quite like a bottle of DRC Richebourg, the 1978 should do nicely: please open and let it breathe for a while, before pouring it extra carefully*.

Anyway: So Menacyn decided to nobble competing dogs and, to do this, Menacyn microbiologists collaborated with in‐house synthetic biologists to create a pathogen that causes *silly behaviour syndrome* ‐ the dogs lose orientation and balance, and fool around more or less like binge drinkers. In anticipation of kennel vets prescribing an effective antibiotic therapy, or HMGov developing a bacterial virus cocktail against the bug, when designing it, Menacyn synbiologists made it resistant to all antibiotics in current therapy and resistant to all phages^14^ they could isolate. You believe it cannot be treated.

To secure a legitimate distribution chain, Menacyn bought up Staines Pet Products, a traditional and well‐respected family‐owned pet food retailer, gave it a minimal make‐over, started to market new, mostly harmless probiotic products, and rapidly developed a countrywide product distribution network. Via this distribution network, it has gained access to most professional dog kennels and is able to selectively target and sabotage racing dogs against which it will bet.

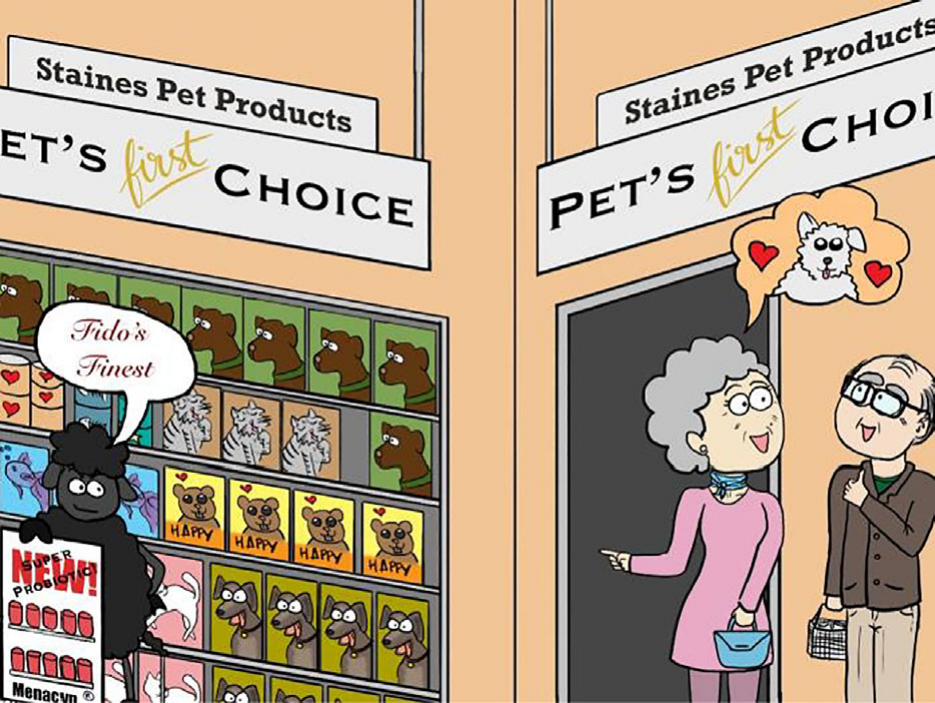

***von‐Spectre****, tossing back another 4 fingers:* James: stop being so dramatic and cut to the chase!

***Bondage****:* So here’s the thing. The *SBS* bug invades the intestinal epithelium and enters the blood stream. Now the dastardly Menacyn researchers had engineered into *SBS* key tissue tropism functions^15^ of a meningitis‐producing pathogen, such that *SBS* homes in on the blood:brain barrier, which it then crosses and sets up a local infection in the brain. The inflammatory response thereby triggered results in loss of bodily control and focus that is characteristic of *SBS*. Well, just as you developed a vaccine to protect the dogs you bet on, our secret high‐tech germ warfare defence group has also developed a super‐effective anti‐*SBS* vaccine that is currently being deployed in greyhound breeding establishments country‐wide. Moreover, it turns out that this group, in collaboration with an ingenious British microbiologist who shall remain nameless to prevent her being, shall‐we‐say, *neutralised* by Menacyn, has been working for quite some time on tissue tropisms of infectious agents – i.e. their tissue homing systems – and has developed some interesting and diverse strategies to sabotage the process of pathogens travelling to the sites where they like to set up shop to do their dirty work. One of these involves new molecules that subvert the normal tropism homing system: a bit like the metal fragment clouds used by warplanes to avoid ground‐to‐air missiles. Another is a completely new type of antimicrobial that targets the synthesis of tropism‐centric cell surface receptors/lectins. They have also collaborated with a world expert on synthetic microbiology to develop a totally synthetic bacterial virus that uses these same receptors to infect the Menacyn bug and kill it. So, in case *SBS* mutates and the vaccine loses protective activity, HMGov has multiple secret options to spike your greyhound infection gun.

***von‐***Spectre*** , sipping the last 4 fingers:*** Well, James, that is an interesting, if implausible story. But on reflection I may mention it in passing to one or two individuals who may have an interest. In the unlikely event that they or people they know wish to contact HMGov, I’ll request another meeting with you via our usual dead drop.

***Bondage:*** Sure, old sport. Of course, next time we meet, Menacyn will have to use some of its ill‐gotten gains to pick up the tab, which will certainly include a good bottle from the DRC.

***Part 4: A week later, 9.34 pm, Southend High Street, just exiting from the Acropolis fish and chips bar and on the way to The Hound and Hare***

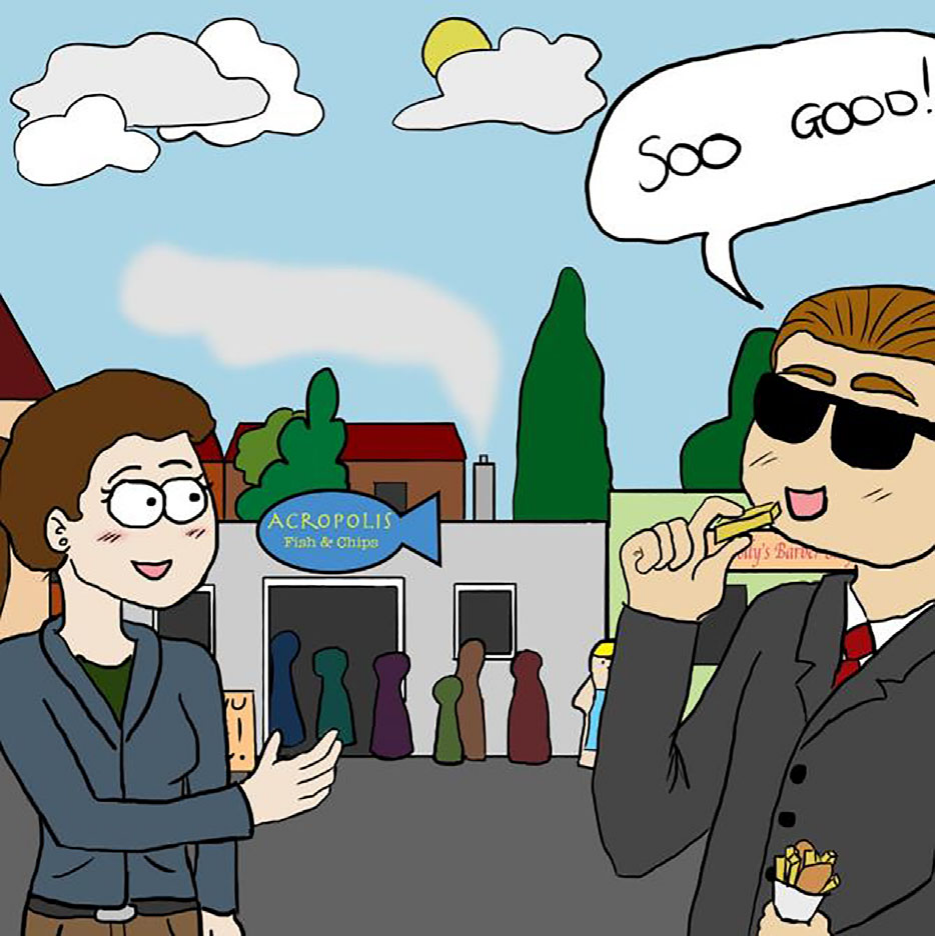

***Bondage, munching chips with a look of intense pleasure:*** These are SOO good! Well Jill: that was nice teamwork! And the DRC was outstanding, if a little extravagant for the occasion – good job Menacyn was paying! I don’t think we’ll have any more *SBS* problems, though I cannae promise that Menacyn won’t come up with a new wheeze to make money.

***Jilly:*** James: it was such fun connecting with you again, and seeing Menacyn, and especially that awful von Spectre snake, frustrated in their efforts to corrupt the nice clean sport of dog racing. Fortunately, *Super Bdello* was super successful in dealing with *SBS* in the poor infected dogs, and that bit of research I could do quickly on the side on the development of new ligands that sabotage brain tropisms of pathogens would seem to provide a long term solution to existing and yet‐to‐be‐developed pathogens that home in on the blood:brain barrier.

***Bondage*** : Aye, and you should have seen the look on Mam’s face when I told him that – he looked as though he had just swallowed a lump of freshly‐egressed camel dung!

***Jilly:*** Actually, James, after the work on new ligands, I started a project on tissue tropism which may be interesting for you and MI57. I don’t know if you know this but the nature of tissue tropisms of infectious agents was described by the famous microbiologist, Harry Smith.^19^ In his work on *Brucella abortus*, a nasty and contagious bug that infects the bovine placenta and causes abortion, Harry showed that the placenta contains a special sugar, erythritol, which is not present in significant quantities anywhere else in the body. He further showed that *B. abortus* needs erythritol for growth: ergo the erythritol basis of the tropism of *B. abortus* for the placenta^16^. Since that time, many infectious agents have been shown to exhibit tissue or cellular tropisms, including of course meningitis‐causing bugs, like *Neisseria meningitidis*, which target the meninges, HIV, which specifically infects CD4 T‐cells, etc.^15^, ^16^ Interestingly, the property of tissue tropism is not all bad news since some pathogens, like *Salmonella* and *Newcastle Disease Virus,* specifically target tumour cells and are being explored as a platform for new potential anti‐cancer therapeutic agents.^17^

***Bondage****:* Aye: every cloud has a silver lining!

***Jilly:*** Absolutely! At the mo, I am having such fun collaborating with Vic Torde of the Lorenzo von Syntech High Security Institute for Artificial Life in Madrid^18^ on a variety of projects, mostly to do with the production of synthetic decoys^17^, molecules that mimic the surface receptors of our cells – the cellular doors – onto which pathogens dock in order to enter and cause mischief in them. When delivered by an appropriate route in an infected person, molecular decoys have the potential to neutralise a pathogen, which attaches to the decoys instead of our cells. This prevents the body being overwhelmed before the immune system can get its act together.

***Bondage****:* Now that sounds really interesting: I think our germ warfare defence folk at Porton Up! (see also: https://www.bbc.com/news/uk‐48540653) will be contacting you again to discuss this.

***Jilly:*** Oh, that will be nice! Porton Up! has pioneered such a lot of key work on microbial pathogenesis and I really enjoyed working with them on *SBS*.

In fact, molecular decoys may well become a significant weapon in our armoury against infectious diseases, given the antibiotic resistance crisis^20^, which is increasingly rendering physicians helpless when confronted with previously treatable infections, particularly nosocomial infections.

And you know: molecular decoys are in discussion as therapies for COVID‐19^21^. The virus causing the pandemic, SARS‐CoV‐2, attaches via its viral surface spike protein to a cell surface receptor named angiotensin‐converting enzyme 2, or ACE2 for short^22^, ^23^, ^24^.

***Bondage****:* Aye: I have heard about *long COVID* *^24^*: it sounds grim!

***Jilly:*** Yes, the SARS virus is really nasty, so anything we can do to try and sabotage it is worthwhile. Vic and I are trying to make a contribution to this by using synthetic microbiology to programme a well‐known bacterial cell factory called *Pseudomonas* KT2440^25^ to produce different decoys that are non‐immunogenic and have no biological activity, and that efficiently attach to the SARS‐CoV‐2 spike protein and block it from binding to ACE2. If we can find a good decoy, it might become a useful prophylaxis and therapy agent: in future, you may be able to pop along to a pharmacy and buy a nasal spray called Pseu‐dec that will protect you from any virus that uses the ACE2 receptor.

***Bondage****:* Well, that would be wonderful because, as far as I know, we do not yet have any good anti‐viral drugs effective against COVID!

***Jilly:*** Exactly! And of course, if this works well, it could serve as a generic platform for the production of similar products with different decoys that protect against other respiratory pathogens. Decoys could become a new first‐line defence in dealing with pandemics, while scientists are busy developing a vaccine.

***Bondage****:* Well, our response to the *SBS* story has certainly triggered some very interesting science that may lead to new prevention‐treatment products.

***Jilly:*** Definitely! And by the way, decoys are nothing new in biology. In fact they are standard weapons in the ecological predator:prey relationships. Disguise is yet another strategy! For example, some streptococci – the bugs that cause sore throat ‐ can bind and cover themselves with various host proteins, like fibronectin, and thereby disguise themselves as host cells, which are not then recognised and attacked by host defences^26^.

And such stratagems are not only found in human and animals pathogens: plant pathogens have evolved decoys to sabotage plant defences against infection^27^. But savvy microbiologists will try to turn the tables on microbial use of decoys by using synthetic microbiology to create new decoys that frustrate pathogens. Actually, decoys against plant pathogens might be a good idea for the next project Vic and I start! This may also be something Porton Up! should look at because evil bioterrorism might come across the idea of focusing on food security in future.

***Bondage****:* Well, lass: I am glad you told me that, because MIH57 always tries to be one step ahead of the villains of this world. And it is pretty obvious that wily microbiologists like you will be essential to our efforts to stymie some villain’s evil plan.

***Jilly:*** Oh, I’d love to get involved again in one of your future missions to save Britain from attack. I have a number of other microbial tricks up my sleeve. And it would also be incredible to sample one of those exquisite but, for an academic, unaffordable DRCs sometime: they are bio‐wines, you know (https://www.decanter.com/features/decanter‐man‐of‐the‐year‐aubert‐de‐villaine‐246429/)

**Dedication:** This Crystal Ball is dedicated to the memory of Singh Chhatwal^28^, who pioneered research on disguises and decoys produced by *Streptococcus*, and so much more.

## Explanatory Notes

^1^ This is the second yarn in the Bondage series; ^9^ was the first. To view other issues of *Microbiome Yarns*, go to http://www.theabsurdmicrobe.com/discoveries‐oldstuff/

^2^ Health Warning. This article is a figment of the authors’ imagination; any serious contemplation may unnecessarily challenge the mental harmony of the reader. Nevertheless, some of the ideas floated in it may be relevant to future research endeavours, and hence it constitutes a Crystal Ball.

^3^ any possible resemblance of the fictional characters and organisations in this fable to a real person, organisation or enterprise is coincidental and unintentional

^4^ Dedicated to pioneering researchers of *Bdellovibrio*, tissue tropisms of pathogenic microbes and their underlying mechanisms, especially Harry Smith, molecular decoys and disguises, and to those heroes of scientific humour, especially Dan Koshland (e.g. Koshland, D. E., Jr. 1994. Editorial: A simple proposal for health care rationing. *Science* **264**: 1515) and David Jones (e.g. Jones, D. 1999. Daedalus: Internal ecology. *Nature* **402**: 367.), who have sadly left us to join other scientific greats in that ethereal elite university in the sky, where they continue to turn scientific discoveries into humorous fantasies with more than a grain of plausibility

^5^ Abject apologies, but in naming this Essex character, I was not able to resist the temptation to weave in a historical staple food – jellied eels – of London and the Eastern Counties (and other regions, of course: it was after all an international dish: https://en.wikipedia.org/wiki/Jellied_eels; https://www.youtube.com/watch?v=9TTGrN4W8nQx)

^6^ Stolp, H. and Starr, M.P. (1963) *Bdellovibrio bacteriovorus* gen. et sp. n., a predatory, ectoparasitic, and bacteriolytic microorganism. *Ant V Leeuwenhoek* **29**: 217–248; Negus, D., Moore, C., Baker, M., et al (2017) Predator versus pathogen: how does predatory *Bdellovibrio bacteriovorus* interface with the challenges of killing Gram‐negative pathogens in a host setting? *Annu Rev Microbiol* **71**: 441‐457.

^7^ The wonderful image of *Bdellovibrio*, given false colour drama by GM, was kindly provided by Auxi Prieto and shows *Bdellovibrio* on the left and *Bdellovibrio* giving short shrift to *Pseudomonas* KT2440 on the right; Martinez, V., Herencias, C., Jurkevitch, E. and Prieto, A. (2016) Engineering a predatory bacterium as a proficient killer agent for intracellular bio‐products recovery: the case of the polyhydroxyalkanoates. *Sci Rep* **6**: 24381; Gonzalez, E., Herencias, C. and Prieto, A. (2020) A polyhydroxyalkanoate‐based encapsulating strategy for ‘bioplasticizing’ microorganisms. Microb Biotechnol **13**: 185‐198; Bagdasarian, M.R. Lurz, B. Rückert, et al (1981). Specific‐purpose plasmid cloning vectors. II. Broad host range, high copy number, RSF1010‐derived vectors, and a host‐vector system for gene cloning in *Pseudomonas*. *Gene* **16**: 237–247.

^8^ van Sorge, N.M., Quach, D., Gurney, M.A., et al (2009) The Group B Streptococcal serine‐rich repeat 1 glycoprotein mediates penetration of the blood‐brain barrier. *J Infect Dis* **199**: 1479–1487; Banerjee, A., Kim, B.J., Carmona, E.M., et al (2011) Bacterial pili exploit integrin machinery to promote immune activation and efficient blood‐brain barrier penetration. *Nature Comm* **2**: 462

^9^ see Timmis K. and Jebok F. (2018) Microbiome Yarns: human milk oligosaccharides, *Bifidobacterium* and immunopowergames. *Microb Biotechnol* **11**: 437–441.

^10^ a comprehensive reorganisation of MI6 (Military Intelligence Section 6), done in the British style, following exposure of many of its operatives by Wackidribbles and subsequent hacking attacks from 8‐year old children at a primary school in Aldershot, resulted in its breakup into multiple small, unconnected cells with MI designations reflecting favourite military meals. James Bondage is the star of MIH57, the counter‐espionage unit charged with the most difficult and dangerous operations.

^11^ Masters of the Universe: see *The Bonfire of the Vanities* by Tom Wolfe

^12^ World’s Best Vodka (*tasting notes: understated with a savoury broad backdrop; crisp slightly grainy notes, subtle, refined and lifted and layered on the nose, suggestive of baked bread, cereal, sherbet and spice; elegantly tight on the palate with poise, restraint and balance; graceful texture and subtle complexity; rounded in the mouth with delicate flavours and a harmonious structure; long and evolving on the finish; bright and refined with a pronounced focus and persistent finish*); the perfect accompaniment to stressful meetings and excessive video conferences, and ideal enabler of exploration of the diverse states of Pickle Factor, P, of FDIPA^13^.

^13^ Timmis, K., Jebok, F., Molinari, M., Rohde, M. and Lahti, L. (2018). Microbiome yarns: human biome reproduction, evolution and visual acuity. *Microb. Biotech*. **11**: 149–159.

^14^ Brüssow, H. (2017) Infection therapy: the problem of drug resistance – and possible solutions. *Microb Biotechnol* **10**: 1041–1046.

^15^ Orihuela, C.J., Mahdavi, J., Thornton, J., et al (2009) Laminin receptor initiates bacterial contact with the blood brain barrier in experimental meningitis models. *J Clin Invest* **119**: 1638–46 ; Doran, K.S., Fulde, M., Gratz, N., et al (2016) Host–pathogen interactions in bacterial meningitis. *Acta Neuropathol* **131**: 185–209.

^16^ Smith, H, Williams, E., Pearce, J,H., Keppie, J., Harris‐Smith, P.W., Fitzgeorge, B. and Witt, K. (1962) Foetal erythritol, a cause of localization of *Brucella abortus* in bovine contagious abortion. *Nature* **193**: 47–49; Williams, A, E., Keppie, J. and Smith, H. (1962) The chemical basis of the virulence of *Brucella abortus*. III. Foetal erythritol. A cause of the localisation of *Brucella abortus* in pregnant cows. *Br J Exp Pathol* **43**: 530–537; Smith, H., Anderson, J.D., Keppie, J., Kent, P.W. and Timmis, G.M. (1965) The inhibition of the growth of brucellae *in utero* and *in vivo* by analogues of erythritol. *J Gen Microbiol* **38**: 101–108; Dixon, B. (1971) Why do microbes misbehave? New Scientist and Science Journal, September 16 **51**: 625–627; Smith, H. (1990) Pathogenicity and the microbe *in vivo* The 1989 Fred Griffith Review Lecture. J *Gen Microbiol* **136**: 377–383.

^17^ Felgner, S., Pawar, V., Kocijancic, D., et al. (2017) Tumour‐targeting bacteria‐based cancer therapies for increased specificity and improved outcome. *Microb Biotechnol* **10**: 1074–1078; Kalyanasundram, J., Hamid, A., Yusoff, K. and Chia, S.L. (2018) Newcastle disease virus strain AF2240 as an oncolytic virus: A review. *Acta Trop*. **183**: 126‐133.

^18^ Timmis, K., Jebok, F., Rohde, M. and Molinari, G. (2018) Microbiome yarns: microbiome of the built environment, paranormal microbiology and the power of single cell genomics. *Microb Biotechnol* **11**: 575–587.

^19^ Gershoni, J.M. (2008) Molecular decoys: antidotes, therapeutics and immunomodulators. *Curr Opin Biotechnol* **19**: 644–651; Keller, M D., Ching, K L., Liang, F X. et al (2020) Decoy exosomes provide protection against bacterial toxins. *Nature* **579**: 260–264.

^20^ Ventola, C L. (2015) The antibiotic resistance crisis: part 1: causes and threats. *Pharm T*herap **40**: 277‐283.

^21^ Chan, K K., Dorosky, D., Sharma, P. et al (2020) Engineering human ACE2 to optimize binding to the spike protein of SARS coronavirus 2. *Science* **369**: 1261–1265; Cao, L., Goreshnik, I., Coventry, B. et al (2020) De novo design of picomolar SARS‐CoV‐2 miniprotein inhibitors. *Science* **370**: 426‐431; Linsky, T. W., Vergara, R., Codina, N. et al. (2020) De novo design of potent and resilient hACE2 decoys to neutralize SARS‐CoV‐2. *Science* 370: 1208‐1214.

^22^ Hoffmann, M., Kleine‐Weber, H., Schroeder, S., et al (2020) SARS‐CoV‐2 cell entry depends on ACE2 and TMPRSS2 and is blocked by a clinically proven protease inhibitor. *Cell* **181**: 271–280.

^23^ Hamming, I., Timens, W., Bulthuis, M C L. et al (2004) Tissue distribution of ACE2 protein, the functional receptor for SARS coronavirus. A first step in understanding SARS pathogenesis. *J Pathol* **203**: 631–637.

^24^ Brüssow, H. (2020) COVID‐19: from pathogenesis models to the first drug trials. *Microb Biotechnol* **13**: 1289–1299; Leung, T Y M., Chan, A Y L., Chan, ER W. et al (2020) Short and potential long‐term adverse health outcomes of COVID‐19: a rapid review. *Emerg Microb Infect* **9**: 2190–2199.

^25^ Nikel P I, de Lorenzo V. (2018) *Pseudomonas putida* as a functional chassis for industrial biocatalysis: From native biochemistry to trans‐metabolism. *Metab Eng* **50**:142–155.

^26^ Hammerschmidt, S., Talay, S R., Brandtzaeg, P. and Chhatwal, GS. (1997) SpsA, a novel pneumococcal surface protein with specific binding to secretory immunoglobulin A and secretory component. *Mol Microbiol* **25**:1113–1124; Medina, E., Molinari, G., Rohde, M., et al (1999) Fc‐mediated nonspecific binding between fibronectin‐binding protein I of Streptococcus pyogenes and human immunoglobulins. *J Immunol* **163**: 3396–402; Fagan, P K., Reinscheid, D., Gottschalk, B. and Chhatwal, G S. (2001) Identification and characterization of a novel secreted immunoglobulin binding protein from Group A Streptococcus. *Infect Immun* **69**: 4851––857; Dinkla, K., Rohde, M., Jansen, W T M et al (2003) Streptococcus pyogenes recruits collagen via surface‐bound fibronectin: a novel colonization and immune evasion mechanism. *Mol Microbiol* **47**: 861‐869.

^27^ Paulus, J K., Kourelis, J. and van der Hoorn, R A L. (2017) Bodyguards: pathogen‐derived decoys that protect virulence factors. *Trends Plant Sci* **22**: 355–357; Paulus, J K. and van der Hoorn, R.A.L. (2018) Tricked or trapped – two decoy mechanisms in host‐pathogen interactions *PLoS Pathog* **14**: e1006761.

^28^ Timmis, K.N. (2016) Singh Chhatwal: a magical scholar and exceptional *Streptococcus* researcher. *Environm Microbiol Rep* **8**: 550–552.

